# Cortical Patterns of Pleasurable Musical Chills Revealed by High-Density EEG

**DOI:** 10.3389/fnins.2020.565815

**Published:** 2020-11-03

**Authors:** Thibault Chabin, Damien Gabriel, Tanawat Chansophonkul, Lisa Michelant, Coralie Joucla, Emmanuel Haffen, Thierry Moulin, Alexandre Comte, Lionel Pazart

**Affiliations:** ^1^Laboratoire de Neurosciences Intégratives et Cliniques, EA 481, Université Bourgogne Franche-Comté, Besançon, France; ^2^INSERM CIC 1431, Centre d’Investigation Clinique de Besançon, Centre Hospitalier Universitaire de Besançon, Besançon, France; ^3^Plateforme de Neuroimagerie Fonctionnelle et Neurostimulation – Neuraxess, Centre Hospitalier Universitaire de Besançon, Université Bourgogne Franche-Comté, Besançon, France

**Keywords:** high density EEG, EEG, emotion, music, chills, musical reward, peak pleasure, cerebral activity

## Abstract

Music has the capacity to elicit strong positive feelings in humans by activating the brain’s reward system. Because group emotional dynamics is a central concern of social neurosciences, the study of emotion in natural/ecological conditions is gaining interest. This study aimed to show that high-density EEG (HD-EEG) is able to reveal patterns of cerebral activities previously identified by fMRI or PET scans when the subject experiences pleasurable musical chills. We used HD-EEG to record participants (11 female, 7 male) while listening to their favorite pleasurable chill-inducing musical excerpts; they reported their subjective emotional state from low pleasure up to chills. HD-EEG results showed an increase of theta activity in the prefrontal cortex when arousal and emotional ratings increased, which are associated with orbitofrontal cortex activation localized using source localization algorithms. In addition, we identified two specific patterns of chills: a decreased theta activity in the right central region, which could reflect supplementary motor area activation during chills and may be related to rhythmic anticipation processing, and a decreased theta activity in the right temporal region, which may be related to musical appreciation and could reflect the right superior temporal gyrus activity. The alpha frontal/prefrontal asymmetry did not reflect the felt emotional pleasure, but the increased frontal beta to alpha ratio (measure of arousal) corresponded to increased emotional ratings. These results suggest that EEG may be a reliable method and a promising tool for the investigation of group musical pleasure through musical reward processing.

## Introduction

The power of music over human emotions is intriguing and there is an ongoing debate regarding not the mechanisms of how music can provoke pleasure but rather why music can be a rewarding experience (Goupil and Aucouturier, 2019). Music causes both marked changes in an individual’s emotional state to the point of musical thrill (Goldstein, 1980) and collective emotional contagion in a social context (Egermann et al., 2011). At the individual level, intense musical emotion involving areas of the limbic system can provoke a pleasurable rewarding experience: the musical chill (Blood and Zatorre, 2001; Salimpoor et al., 2009, 2011). In their paradigm, the participants provided highly pleasurable musical extracts and continuously reported their felt emotional pleasure while listening to music (neutral, low pleasure, high pleasure, and chills). The phenomenon of pleasure associated with chills is composed of two phases, an anticipation phase before the chill when the pleasure is growing, with a specific dopamine release in the dorsal striatum (caudate), and a peak pleasure phase with a dopaminergic release in the ventral striatum (nucleus accumbens) (Salimpoor et al., 2011). In addition, a pharmacological study by Ferreri et al. (2019) demonstrated that dopaminergic releases were not only the consequence but actually one of the causes of the felt emotional pleasure. These cerebral investigations remain limited to laboratory experiments with heavy neuroimaging techniques (fMRI, PET scan) whereas social neurosciences, and consequently the study of collective emotions, is moving toward natural/ecological paradigms (Acquadro et al., 2016; Volpe et al., 2016; Dikker et al., 2017; Bevilacqua et al., 2018; Soto et al., 2018; Swarbrick et al., 2018; Matusz et al., 2019). The use of mobile wireless EEG could provide the possibility of studying cerebral activity during peak emotion of musical chills in ecological/natural conditions, especially because it can be used in hyperscanning paradigms with several participants simultaneously (Lindenberger et al., 2009; Müller et al., 2013, 2018; Sänger et al., 2013; Acquadro et al., 2016).

Electroencephalography (EEG) is a direct measure of electrical activity of the brain that confers a high temporal resolution in the millisecond range. With high-density EEG (HD-EEG), a large area of the scalp is covered by electrodes, which makes it possible to investigate the cortical sources of the surface activity with an acceptable spatial resolution (estimated in the range of a centimeter). Although musical chills have not yet been studied by EEG, HD-EEG enables the source reconstruction to identify the cortical origins of surface activities (Michel and Brunet, 2019). The activities of these sources can therefore be compared with activities already known in structures implicated in musical reward.

Earlier data from classic EEGs suggest that low frequencies such as theta oscillations are involved in reward processing (Aftanas and Golocheikine, 2001; Wingerden et al., 2010; Gruber et al., 2013). Theta activity is reported to be linked to memory processing in reward contexts (Gruber et al., 2013; Pu and Yu, 2019). A stronger activity in the theta frequency range was found in fronto-medial sites after rewarding feedback onset (Pornpattananangkul and Nusslock, 2016) and at occipito-parietal and fronto-central electrodes when comparing anticipation of high versus low reward (Steiger and Bunzeck, 2017). In the musical context, theta band changes in the fronto-central area during music appreciation have been reported and also correspond to high emotional arousal (Lin et al., 2010). Compared with neutral musical excerpts, highly pleasant music provokes a higher oscillatory activity in the theta band over fronto-central regions than neutral musical excerpts (Nemati et al., 2019). The theta power increases in the medial frontal area for positive musical stimuli (Omigie et al., 2015) and for pleasant music (Sammler et al., 2007). The more the loops of the anterior cingulate cortex (ACC) and the medial frontal cortex are activated while listening to music, the more the fronto-medial theta power increases (Jäncke et al., 2015). The specific theta/alpha activities relative to the valence of the music (both aversive/negative and attractive/positive) might be mediated by the amygdala, which is a direct modulator of the auditory and orbitofrontal cortex for processing musical emotion (Omigie et al., 2015). It has also been suggested that theta oscillations play a role in the synchronization of temporal and prefrontal structures during musical emotional processing (Lewis, 2005). Furthermore, the overall frontal alpha EEG asymmetry (power difference between right and left hemisphere), which is reported to be a good indicator of the emotional state while listening to music (Schmidt and Trainor, 2001; Davidson, 2004; Harmon-Jones et al., 2010; Ramirez and Vamvakousis, 2012; Vecchiato et al., 2012; Arjmand et al., 2017; Mennella et al., 2017; Ramirez et al., 2018), should be an interesting candidate for the study of cortical patterns involved during musical chills.

The aim of the present study was to use HD-EEG to identify specific cortical patterns that underlie musical chills. Our main hypothesis was that an increased felt pleasure on a scale from neutral (lowest intensity) to chills (maximum intensity) should produce an increase of the theta activity over the fronto-central regions, and a specific pattern of theta activity over the centro-parietal and temporal regions.

## Materials and Methods

### Ethics

The study was approved by an independent ethics committee (CPP Ouest V – Rennes; no. 2018-A01653-52) and follows recommendations from the French Jardé’s Law (Article R1121-1 1 of the French Law Code of Public Health amended by decree 127 no. 2017-884 of May 2017) on non-invasive protocols involving healthy humans. All participants received the information in full both orally and on paper, and signed a written informed consent form before inclusion in the study.

### Participants

The sample for this study consisted of 18 healthy volunteers (11 women, 7 men) with a mean age of 39.7 years (SD 18.3, range 18–73). All were right-handed (Edinburgh Inventory score >50; Oldfield, 1971), were sensitive to musical reward according to the Barcelona Music Reward Questionnaire (Mas-Herrero et al., 2013; Saliba et al., 2016) (BMRQ overall score >65, Hedonic/High-hyper hedonic group from Mas-Herrero et al., 2014), and frequently experienced chills induced by pleasurable music. Ten participants in this sample were amateur musicians (mean of 20.2 years of practice, SD 13.37, range 10–55).

Recruitment was carried out via advertising posters in our university and hospital. Eighty-nine people responded to our request or contacted us directly to participate in this study. Fifty-eight did not fulfill the eligibility criteria (2 scored lower than 65 for the BRMQ, 3 were not exclusively right-handed, 9 did not provide enough chill-inducing extracts, 36 did not respond after their initial request for information/did not send back questionnaires, for 7 we could not program an appointment, and 1 had a haircut incompatible with EEG recording). Eight participants were not re-contacted after their request to participate because the inclusion deadline had passed. Finally, 23 subjects passed a medical examination to confirm they had normal hearing (with audiogram tests), and an absence of neurological or psychiatric disorders. They all signed an informed consent form. Among them, five participants were ultimately not included in the analysis; three participants reported one or zero instances of chills during the experiment, and for two participants not enough EEG epochs free from artifacts were recorded.

### EEG Recordings

EEG signals were recorded using a 256-channel Sensor Net (from Electrical Geodesics) with a NetAmp 300 high impedance amplifier (Electrical Geodesics). Continuous recordings were performed with a high-pass set at 0.1 Hz and a sampling rate of 1000 Hz; all channels were referenced to the vertex (Cz) and impedances were below 50 kΩ. Cartool software (version 3.7) was used to pre-process all EEG data, a notch filter fixed to 50 Hz was applied, data were band-pass filtered between 1 and 30 Hz, and an average re-referencing of all channels was performed.

### Procedure and Behavioral Data

During the listening session, participants were seated in a comfortable chair, kept their eyes closed, and listened via intra-auricular headphones (Earpods) to five chill-inducing extracts (without any restriction regarding musical style or musical trend) that they had provided and three additional neutral extracts selected by experimenters. They were asked to continuously report their felt emotional pleasure by continuously pushing on a four-button response box according to four levels: (1) neutral, (2) low pleasure, (3) high pleasure, and (4) chills (from Salimpoor et al., 2009, 2011).

**FIGURE 1:**
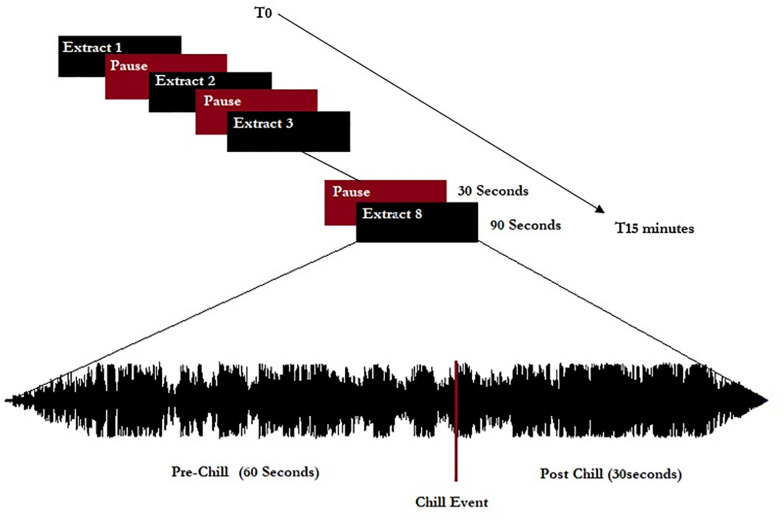
Representation of the experimental procedure. Eight extracts (five chill-inducing extracts and three neutral extracts) were listened to. Extracts were 90 s long and calibrated to start 60 s before indicated chill-inducing moments.

The extracts were separated by 30-s pauses without music, giving a listening session of approximately 15 min total (see schema of experiment in Figure 1). All musical extracts were cut to 90 s, including 60 s before the peak pleasure (indicated by the participants before the experiment themselves), and were normalized to 0 dB including a progressive fade in/fade out of 3 s.

The neutral extracts were selected from a list of musical extracts that had been rated for attractiveness/averseness and arousal by an independent sample of 12 people before the experiment. The mean attractiveness/averseness of the three final selected extracts was between −1 and +1 (on a scale from −5 = “aversive extract” to +5 = “very pleasurable”) and the mean arousal was lower than 3 (on a scale from 0 = “low intensity” to 10 = “max intensity”). The participants included in the study were not familiar with these neutral extracts.

Three listening sessions were recorded using three different EEG systems. Only one recording session used HD-EEG, and the results presented in this paper concern only the HD-EEG data. Participants were asked to report each chill event when they felt high emotional pleasure accompanied by a physical sensation such as goose bumps or thrill, hair standing on end, tingling sensations, or shivers down the spine. If they were already pushing the chills button, they were asked to release and press the chills button again to indicate each “new chill.” Each participant rated their general enjoyment of the experiment (regarding experimental conditions and the experimental procedure) on a 10-point Likert scale from 1 (I did not enjoy the experiment at all) to 10 (I really enjoyed the experiment) and rated each of the own excerpts for arousal (from 1; “this extract is very calming” to 10; “this extract is very exciting/arousing”) and emotional valence (from −5; “this extract has a negative valence, it evoked sad feelings/melancholia, etc.” to +5; “this extract has a positive valence, it evoked joy, happiness etc”).

### Analysis and Statistics

**FIGURE 2:**
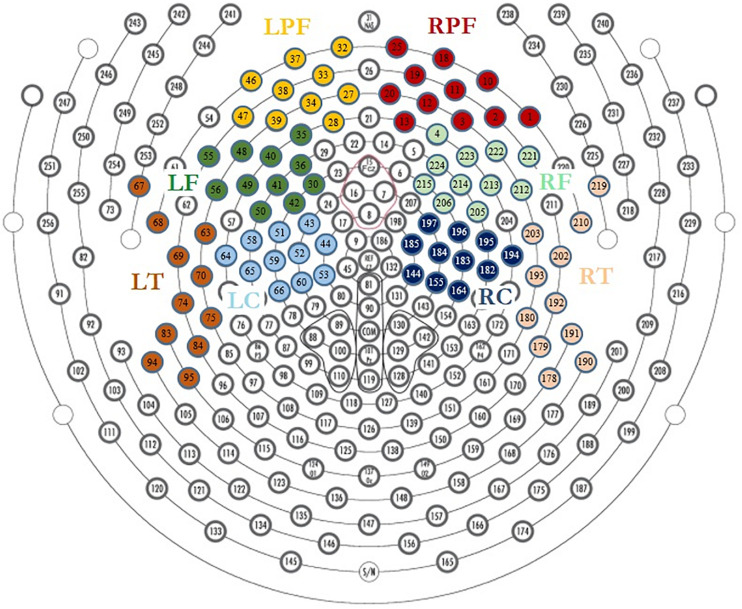
Region of interest for EEG analysis, LPF, left prefrontal; RPF, right prefrontal; LF, left frontal; RF, right frontal; LT, left temporal; RT, right temporal; LC, left central; RC, right central.

The neutral extracts were chosen to be neither pleasant nor unpleasant. Most of the participants reported difficulty distinguishing between the neutral and low pleasure emotional states and usually used only one of the two conditions when reporting low emotional state. These two conditions were therefore grouped together as the “Low pleasure” condition. We extracted the periods of time for which the participant reported each of low pleasure, high pleasure, or chills. Epochs of 1 s were extracted for these periods for the three emotional conditions: (1) low pleasure, (2) high pleasure, and (3) chills. Every epoch with visually detectable artifacts was removed from the analysis. A frequency analysis using fast Fourier transform (FFT) was performed on three frequency bands for each epoch of each condition, theta (4–8 Hz), alpha (8–12 Hz), and beta (12–20 Hz), using MATLAB software (2019a). The power spectral density (PSD) was estimated for each condition using Welch’s method (Hanning window 50% overlap) based on FFT magnitude squared. Eight regions of interest (ROIs) were investigated for each frequency band: left central (LC), right central (RC), left frontal (LF), right frontal (RF), left pre-frontal (LPF), right pre-frontal (RPF), left temporal (LT), and right temporal (RT) (see Figure 2). A calculation of valence was performed following approach/withdrawal theory (Schmidt and Trainor, 2001; Altenmüller et al., 2002; Arjmand et al., 2017); the mean inter-hemispheric PSD value difference in the alpha frequency range was calculated for the frontal area and pre-frontal area as follows:

A⁢l⁢p⁢h⁢a⁢f⁢r⁢o⁢n⁢t⁢a⁢l⁢a⁢s⁢y⁢m⁢m⁢e⁢t⁢r⁢y=(α⁢P⁢S⁢D⁢R⁢F⁢R⁢O⁢I)⁢-⁢(α⁢P⁢S⁢D⁢L⁢F⁢R⁢O⁢I)

A⁢l⁢p⁢h⁢a⁢p⁢r⁢e⁢f⁢r⁢o⁢n⁢t⁢a⁢l⁢a⁢s⁢y⁢m⁢m⁢e⁢t⁢r⁢y=(α⁢P⁢S⁢D⁢R⁢P⁢F⁢R⁢O⁢I)⁢-(α⁢P⁢S⁢D⁢L⁢P⁢F⁢R⁢O⁢I)

An analysis of arousal based on the method used by Ramirez and Vamvakousis (2012) and Ramirez et al. (2018) was performed by calculating the beta to alpha ratio according to the following equation:

$$B\,e\,t\,a\,t\,o\,a\,l\,p\,h\,a\,r\,a\,t\,i\,o=\frac{\left(\beta \,P\,S\,D\,R\,O\,I\,L\,F+\beta \,\mathrm{PSD}\,\mathrm{ROI}\,\mathrm{RF}\right)}{\left(\alpha \,P\,S\,D\,R\,O\,I\,L\,F+\alpha \,\mathrm{PSD}\,\mathrm{ROI}\,\mathrm{RF}\right)}$$

To identify the source that generates the activities observed on the scalp, it is possible to apply source localization analyses that convert EEGs to a real neuroimaging modality (Michel and Brunet, 2019). Based on the structures and regions that are already known to be involved in musical pleasure and music chills, the identification of sources of signal would reinforce the interpretation of surface activities. Source localization was performed on the mean participant’s grand averages of 1-s epochs for each condition. The source localization was estimated using a linear inverse solution based on a local autoregressive average (LAURA) model (Menendez et al., 2001; Brunet et al., 2011). Source localization was applied to five regions of interest, which were created using Cartool software and were based on fMRI literature (Blood and Zatorre, 2001; Brown et al., 2004; Salimpoor et al., 2011): (1) orbito-frontal gyrus (OFG), (2) supplementary motor area (SMA), (3) bilateral insula (Ins), (4) right superior temporal gyrus (RSTG), and (5) left superior temporal gyrus (LSTG).

All statistical analysis was performed using R Studio software (version 3.5.2; 2018-12-20). For behavioral data, Pearson r correlations were performed to explore the link between the number of chills reported and BMRQ scores, sex, age, and years of musical practice. The normality of each data set was confirmed using Kolmogorov–Smirnov tests. For EEG analysis, three conditions (Low pleasure, High pleasure, and Chills) were compared for *alpha frontal asymmetry*, *alpha prefrontal asymmetry*, *beta to alpha ratio*, and for PSD values in the theta and alpha frequency bands for each ROI (LC, RC, LF, RF, LPF, RPF, LT, and RT) using repeated measures ANOVA (α = 0.05) followed by *post hoc* tests using the Bonferroni correction within ROIs. For source localization, repeated measures ANOVA were applied to compare the three conditions within ROIs (SMA, OFC, Ins, LSTG, and RSTG) using grand averages of epochs, and *post hoc* tests were performed using the Bonferroni correction based on the multiplicity of the tests within and between ROIs.

## Results

### Behavioral Data

The 18 participants reported 305 chills during the listening sessions, with a mean of 16.9 (SD 12.8) chills per participant and a mean duration of 8.75 s (SD 10.71) (details in Table 1). The durations of reported chills were very variable across participants. Many chills occurred outside the times of expected peak pleasure. Our analysis revealed no relationship between the number of chill reports and sex, age, BMRQ score, or number of years of music practice. The participants rated their overall enjoyment of the experiment at a mean of 7.9 (SD 0.9, range 5–9) on the 10-point Likert scale. It appears that chills reported during the listening by participants did not always match with the peak pleasure previously indicated by the participant.

**TABLE 1:** Number of reported chills and mean duration by participants.

**Participant**	**Overall BMRQ score**	**Number of reported chills**	**Number of selected musical extracts with at least 1 chill reported (min–max number of chills by extract)**	**Mean duration of reported chills in seconds (SD)**
1	69	15	3 (0–8)	1.40 (0.75)
2	89	17	5 (1–5)	2.14 (1.01)
3	88	19	5 (2–7)	4.22 (6.55)
4	88	16	5 (1–4)	1.72 (0.76)
5	85	19	5 (1–6)	0.2 (0.27)*
6	91	48	5 (5–15)	2.27 (0.78)
7	83	8	5 (1–3)	4.25 (2.41)
8	76	11	5 (1–2)	11.19 (10.21)
9	81	6	5 (1–2)	39.77 (24.29)
10	72	7	4 (1–2)	4.11 (4.67)
11	79	9	5 (1–2)	31.96 (21.76)
12	84	4	4 (1–1)	15.92 (9.54)
13	73	14	5 (2–5)	10.06 (9.09)
14	91	13	5 (1–4)	5.2 (2.02)
15	72	8	2 (1–7)	4.88 (2.19)
16	78	50	5 (8–13)	3.45 (1.48)
17	84	17	5 (2–6)	5.26 (2.60)
18	83	24	5 (1–8)	5.18 (4.49)

*Participants five indicated only the beginning of the felt chills and did not maintain the button pressure.

### EEG Results

#### Power Spectral Density Values

##### Theta frequency band

**FIGURE 3:**
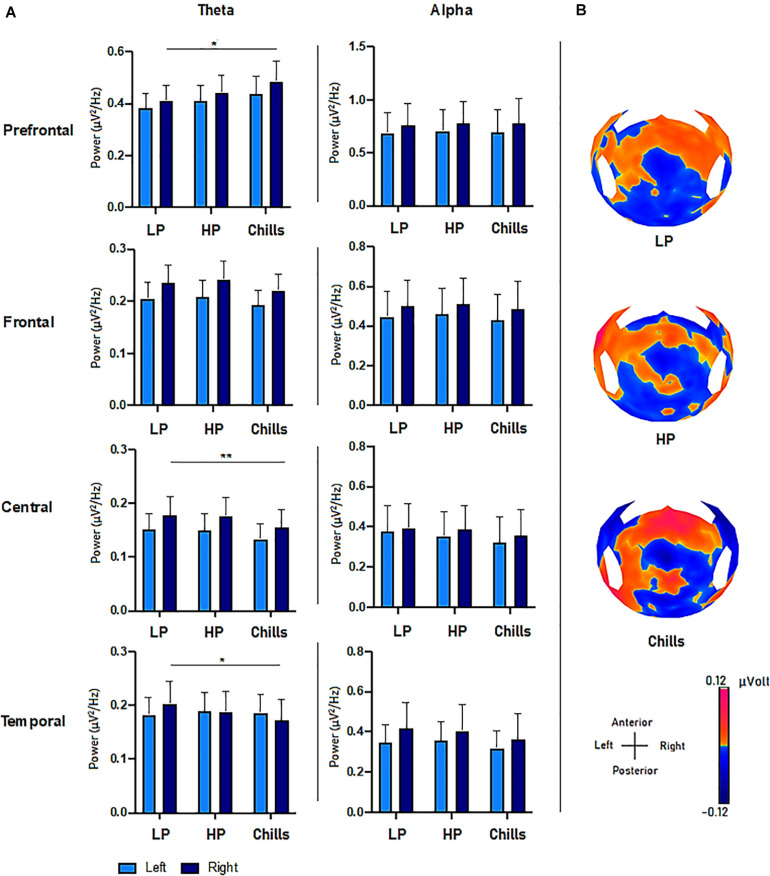
**(A)** Comparison of power spectral density (μV^2^/Hz) values in the theta and alpha frequency range for each condition (LP, low pleasure; HP, high pleasure; Chills) in the prefrontal, frontal, central, and temporal ROIs (*p < 0.05, **p < 0.01, errors bars represent SEM). **(B)** Surface topography for each condition showed an increased positivity for chills and a gradually increasing positivity in parieto-central sites as the emotion increased (μV).

The results show specific activity in the theta frequency range in the central, right prefrontal, and right temporal regions. The comparison of PSD values in the theta frequency range for each condition (Low pleasure, High pleasure, and Chills) revealed a significant group effect for the LC ROI [*F*(2.15) = 3.75; *p* = 0.033], RC ROI [*F*(2.15) = 4.09; *p* = 0.025], RT ROI [*F*(2.15) = 5.88; *p* = 0.006], and RPF ROI [*F*(2.15) = 3.28; *p* = 0.049] (Figure 3). *Post hoc* analysis revealed that PSD values were significantly lower for Chills versus Low pleasure (RC ROI *p* = 0.042; RT ROI *p* = 0.004) and the PSD values were significantly higher for Chills versus Low pleasure for the RPF ROI (*p* = 0.046).

*Post hoc* analysis did not reveal any differences between Chills versus Low pleasure for the LC ROI (*p* = 0.058), or any significant differences between Low and High pleasure for LC ROI (*p* > 1), RC ROI (*p* > 1), RT ROI (*p* = 0.3), and RPF ROI (*p* = 0.86). *Post hoc* analysis also did not reveal significant differences between High pleasure and Chills for the LC ROI (*p* = 0.085), RC ROI (*p* = 0.072), RT ROI (*p* = 0.27), or RPF (*p* = 0.44).

There were no significant effects between the different conditions but rather “tendencies” showing an increase in the theta PSD value for the LPF ROI [*F*(2.15) = 2.84; *p* = 0.071], and a decrease in the theta PSD value for the LF ROI [*F*(2.15) = 2.78; *p* = 0.075] and RF ROI [*F*(2.15) = 3.15; *p* = 0.055] as the emotional rating increased (Figure 3). There were no effects for the LT ROI [*F*(2.15) = 0.13; *p* = 0.87].

##### Alpha frequency band

The same analysis comparing the PSD value in alpha frequency bands for each condition did not reveal significant group effects for any ROI (all *p*-values > 0.9) (Figure 3).

#### Beta to Alpha Ratio and Alpha Asymmetry

**FIGURE 4:**
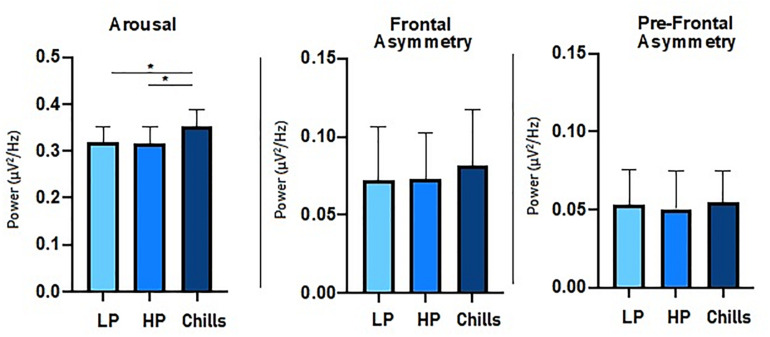
Comparison of power spectral density (μV^2^/Hz) value for each condition (LP, low pleasure; HP, high pleasure; Chills) for *beta to alpha ratio*, *frontal asymmetry*, and *prefrontal asymmetry* (*p < 0.05, errors bars represent SEM).

For *beta to alpha ratio* analysis, the comparison of each condition showed a significant group effect [*F*(2.15) = 4.77; *p* = 0.014] (Figure 4). *Post hoc* analysis revealed significant differences between Chills and the two other conditions (Chills vs. Low Pleasure, *p* = 0.041; Chills vs. High Pleasure, *p* = 0.028). The *beta to alpha ratio* was higher for chills than for low pleasure and high pleasure.

For asymmetry analysis, the comparison of each condition did not show a significant group effect either for *alpha frontal asymmetry* [*F*(2.15) = 0.32; *p* = 0.75] or for *alpha prefrontal asymmetry* [*F*(2.15) = 0.06; *p* = 0.94] (Figure 4).

#### Source Imaging

**FIGURE 5:**
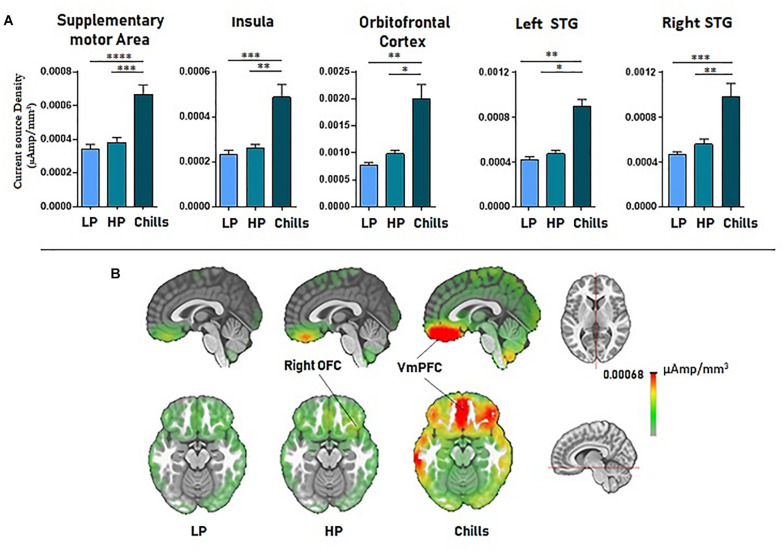
**(A)** Current source density (μA/mm^3^) for each condition (LP, low pleasure; HP, high pleasure; Chills) for the supplementary motor area, bilateral insula, orbitofrontal cortex, and superior temporal gyri. **(B)** Representation of cortical activations (μA/mm^3^) for the orbito-frontal cortex/VmPFC complex in the sagittal and the transversal planes (*p < 0.01, **p < 0.001, ***p < 0.0001, ****p < 1.10^–5^, errors bars represent SEM).

Source localization revealed a positive link between increased emotional rating and intensity of activation of the medial orbito-frontal cortex [*F*(2.15) = 17.4; *p* < 1.10^–5^], the bilateral insula [*F*(2.15) = 21.63; *p* < 1.10^–6^], the supplementary motor area [*F*(2.15) = 27.3; *p* < 1.10^–7^], and the left and right superior temporal gyri [LSTG: *F*(2.15) = 17.76, *p* < 0.00001; RSTG: *F*(2.15) = 22.05, *p* < 1.10^–6^]. Statistical analysis revealed a significant difference for Chills versus Low pleasure (OFC: *p* < 0.001, Ins: *p* < 0.0001, SMA: *p* < 1.10^–5^, LSTG: *p* < 0.001, RSTG: *p* < 0.0001) and for Chills versus High pleasure (OFC: *p* < 0.01, Ins: *p* < 0.001, SMA: *p* < 0.0001, LSTG: *p* < 0.01, RSTG: *p* < 0.001) (see Figure 5).

There were no significant differences when comparing all the ROIs for High versus Low pleasure (all *p*-values > 1).

## General Discussion

The aim of the present study was to investigate the neural processing that occurs during musical chills using HD-EEG. The EEG results confirmed a specific activity over the prefrontal regions in the theta frequency band, but we observed no specific alpha asymmetry patterns relating to the approach/withdrawal system. Source localization allowed us to identify greater activities in the insula, OFC, and SMA, which confirmed the specificity of surface activity for chills. These three activated areas are in line with previous findings of neuroimaging studies that also identified the bilateral insula, OFC, ventromedial prefrontal cortex, SMA, and ventral/dorsal striatum involved in musical chills (Blood et al., 1999; Blood and Zatorre, 2001; and Brown et al., 2004).

The specific theta activity has been previously identified in musical emotional processing (Ramos and Corsi-Cabrera, 1989). The results from the prefrontal areas confirm previous findings that suggested a higher theta band activity over the fronto-midline regions and fronto-medial region for pleasant versus neutral musical excerpts (Sammler et al., 2007; Jäncke et al., 2015; Nemati et al., 2019), for positive musical stimuli (Omigie et al., 2015; Rogenmoser et al., 2016). Theta activity might be linked with reward anticipation, memory, and attention (Burgess and Gruzelier, 1997; Krause et al., 1999; Gruber et al., 2013; Pu and Yu, 2019), which is a key component of emotional responses to music (Brattico and Pearce, 2013). More precisely, theta activity is associated with successful memory performance in context of high reward and is reported to be linked with dopaminergic activity (Gruber et al., 2013). The specific frontal theta activity could be the surface activity reflection of the reward system activation via a network including the amygdala, insula, and OFC, which has previously been found to correspond to the occurrence of chills (Blood and Zatorre, 2001). The representation of source activity also suggests that the ventral tegmental area is involved during the chills, despite it not being one of the structures we targeted in our analysis. The OFC is involved in reward processing (Berridge, 2003) and is strongly connected with reward structures such as the amygdala, parahippocampal cortex, and medial prefrontal cortex (Bechara et al., 1994). It is implicated in the system underlying autonomic responses to music (Koelsch, 2014), and activation of the right OFC has been correlated with pleasantness ratings (Blood et al., 1999) and intensity of chills (Blood and Zatorre, 2001). It has been established that the intensity of chills is correlated with the intensity of striatum and OFC activity (Blood and Zatorre, 2001; Salimpoor et al., 2011) and that theta activity could be a cortical activity related to reward structure activation. Considering these facts in addition to the gradual increase of theta activity in the prefrontal ROI when the emotional ratings increased, we can reasonably hypothesize that the increased power of the theta activity is linked to the intensity of the felt pleasure. Further theta activity over fronto-central sites might be dependent on arousal level (Sammler et al., 2007). As suggested by works from Jäncke et al. (2015), the increased activity in the right versus left OFC that was observed with source localization corresponded to the significant right prefrontal increased theta activity. It is also one more argument in favor of the implication of the OFC in the production of theta prefrontal activity. However, the same overall trends found in both the left and right prefrontal areas suggest that musical pleasure is reflected on both left and right frontal sites, independent of positive or negative emotional valence.

The SMA is known to be associated with the reward system (Knutson et al., 2001), and its involvement has previously been identified during musical chills using PET; the rCBF increased in the SMA during chills (Blood and Zatorre, 2001). Furthermore, a correlation analysis demonstrated that the rCBF in the SMA (as well as the insula and the OFC) was positively correlated with increase in several psychophysiological parameters (HR, respiration rate, and electromyography). Decreased theta oscillatory activity in the central region may be related to the specific SMA activity during chills. Both source localization and surface activity showed a large difference for chills compared with the two other conditions, and there were no differences between low versus high pleasure. At this point, we are not able to explain the stronger effect of theta decrease in the right compared with the left central ROI, even though—as for the prefrontal areas—the same trends were found in both left and right ROIs.

The results of our study also highlighted a gradual decrease of theta power for the right temporal ROI and an increased activity in both the left and the right superior temporal gyrus. Because the superior temporal gyrus is known to be activated in the processing of musical liking/disliking (Brown et al., 2004), and familiarity influences neural responses in the auditory cortex (Pereira et al., 2011), we hypothesized that this temporal activity is related to the auditory cortex. The auditory cortex localized in the superior temporal gyrus is connected to the OFC, and these two structures exchange information during music processing (Brown et al., 2004). The activity, which was identified with source localization from the right superior temporal gyrus by Joucla et al. (2018), is likely to be a signature of music liking, and these findings seem to be consistent with ours. Furthermore, a study predicted that people who frequently experience musical chills have a higher structural connectivity (larger volume of white matter connectivity) between the prefrontal medial cortex, insula, and posterior superior temporal gyri (Sachs et al., 2016). We suggest that the theta activity in the right temporal ROI could be related to music liking processing. However, we observed increased activation of both the bilateral insula and the left and right temporal gyri with higher emotional ratings. It is difficult to draw a clear conclusion about the right temporal surface activity because the insula and the auditory cortex are adjacent structures that could both have been involved.

We found no specific effect both for oscillatory rhythms in the alpha band or for alpha frontal and prefrontal asymmetry. We hypothesized that the climax of an emotional increase—related to the activation of the approach dimension relative to positive/rewarding stimulation (Omigie et al., 2015)—from neutral to chills should gradually activate the approach dimension, resulting in increased alpha asymmetry. The extracts for each participant were not restricted to only positive or negative valence, and this should have affected the asymmetry results. The major or minor mode of the music can transmit happy or sad emotions, which, respectively, increase or decrease the alpha power in the left frontal area (Schmidt and Trainor, 2001; Tsang et al., 2001). The experience of chills is quite difficult to reproduce on demand so we did not restrict the valence of the stimuli because this was neither an aim nor would have been a gain for these investigations. We considered that such a restriction was not in line with our perspectives and would have reflected the cerebral activity relative to only positively or negatively valenced pleasurable musical chills.

The arousal calculation showed an increased β/α ratio on frontal regions. There was no effect on the alpha band over the frontal region; however, the same non-significant trends can be identified for most of the participants, who exhibited decreased alpha power on the frontal ROI during chills, which explains our results. Several structures were identified using fMRI, such as the amygdala, prefrontal cortex, or even auditory cortex, which were linked with arousal changes while listening to music (Daly et al., 2019). Further connectivity analysis using fMRI has already demonstrated a close relationship between the nucleus accumbens, ventral tegmental area, and hypothalamus in investigations of affective responses to music (Menon and Levitin, 2005). Connectivity analysis also highlighted a close relationship between several structures of the affective and autonomic systems. The hypothalamus and particularly the insula, which showed connectivity with the nucleus accumbens during music listening (Menon and Levitin, 2005), corresponds to the sources localized in our study.

There are some limitations to our experiment. Because of experimental constraints, the chills might have been “auto-induced” because participants expected the specific chill-inducing moment that they had previously indicated. This could have reinforced the anticipation phase highlighted by Salimpoor et al. (2011). Thus, the investigation of the chills induced by music in a natural ecological setting could provide somewhat different EEG results. Furthermore, several participants reported that the “aseptic” environment of our experimental room could influence the emotions, and thus the experience of chills. For this reason, some participants did not report enough chills and were therefore excluded from EEG analysis. More occurrences of chills per subject, as well as a larger sample, would have reinforced the overall EEG results and the robustness of effects. The influence of the headphones on the local electric field has not been evaluated and the motor activity related to continuous reports might have slightly influenced the oscillatory activity.

## Conclusion

In conclusion, our results suggest that HD-EEG could provide relevant information about musical pleasure and pleasurable musical chills mainly in the right prefrontal, temporal, and central areas in the theta band. This work is a first step for the study of musical chills using EEG in more ecological paradigms. In future experiments, with the development of wireless mobile EEG systems and coupled with physiological measures, EEG recordings characterizing musical pleasure and musical chills in both social contexts and live conditions would provide a relevant parameter to study emotional synchronization of groups (Chabin et al., 2020).

## Data Availability Statement

The datasets presented in this study can be found in online repositories at the following address: https://figshare.com/articles/Cerebral_activity_during_peak_emotion_in_response_to_music_revealed_by_High-Density_EEG_/11687868.

## Ethics Statement

The study was approved by an independent ethics committee (CPP Ouest V – Rennes; no. 2018-A01653-52) and follows recommendations from the French Jardé’s Law (Article R1121-1 1 of the French Law Code of Public Health amended by decree 127 no. 2017-884 of May 2017) on non-invasive protocols involving healthy humans. All participants received the information in full both orally and on paper, and signed a written informed consent form before inclusion in the study.

## Author Contributions

TChab, AC, DG, and LP: conceptualization. TChab, CJ, LM, and LP: investigations. TChab and TChan: data curation. TChab and DG: formal analysis. TChab, DG, and LP: writing–original draft. TChab, AC, DG, EH, TM, and LP: writing–review and editing. All authors contributed to the article and approved the submitted version.

## Conflict of Interest

The authors declare that the research was conducted in the absence of any commercial or financial relationships that could be construed as a potential conflict of interest.
